# Synthesis of Homogeneously {100}-Textured 3-Inch Free-Standing Diamond Wafer

**DOI:** 10.3390/ma19112398

**Published:** 2026-06-04

**Authors:** Jing Zhang, Stephan Handschuh-Wang, Zhicheng Xing, Tao Wang

**Affiliations:** 1 School of Integrated Circuits, Shenzhen Polytechnic University, Shenzhen 518055, China; 2 Glory Zenith Jinxel Semiconductor Co., Ltd., Shenzhen 518110, China; 3 College of New Materials and New Energies, Shenzhen Technology University, Shenzhen 518118, China; 2410262078@stumail.sztu.edu.cn

**Keywords:** {100}-textured microcrystalline diamond, nitrogen addition, MPCVD, diamond, large area

## Abstract

A two-step growth process was developed to fabricate a 3-inch homogeneously {100}-textured free-standing diamond wafer by microwave plasma enhanced chemical vapor deposition (MPCVD). The sequential growth process is based on a change in the growth parameter α, which is given by the growth rates on {100} and {111} facets, α = $\sqrt{3}$·(V_100_/V_111_). Initial growth was executed with nitrogen addition, yielding an α value close to 3 for evolutionary selection of the (100) face, followed by cessation of nitrogen addition to yield a lower α value. A homogeneously grown {100}-textured diamond over an area of ca. 175 cm^2^ with a thickness ≥ 0.8 mm was obtained after 196 h growth. The diamond growth rate was 4.0–5.5 µm/h, which is four times higher than the conventional growth of oriented diamond. This was substantiated by optical microscopy, Raman spectroscopy, and XRD analysis. The large crystal size of 210 ± 60 µm has been assigned to the second growth step, where growth is preferentially in the <111> direction. The homogeneous {100} texture and the large crystal size are conducive to achieving high thermal conductivity, as the in-plane thermal conductivity of the polycrystalline diamond wafer was increased from ca. 850 W/(mK) to 1125 W/(mK).

## 1. Introduction

Due to the extraordinary thermal, electrical, and mechanical properties of diamond, a variety of research and commercial applications have been established [1]. For instance, single crystal diamond (SCD) is considered the pinnacle for thermal management (i.e., heat spreader [2,3]) due to the extraordinarily high thermal conductivity of ca. 2200 W/(mK) at room temperature [4]. In contrast, the thermal conductivity is reduced for polycrystalline diamond due to the grain boundaries, and the reduction is exacerbated by a reduction in grain size [5]. This reduction is associated with abundant phonon scattering events and reduced phonon mean free path [6]. This makes SCD an interesting candidate for thermal management applications. However, high-quality large-area SCDs are difficult to produce because of limitations related to size and high-quality seed crystals [7], as well as their scarcity and high cost, and economic viability (value proposition vs. cost). Alternatively, the growth of textured diamond coatings can avoid the degradation of thermal conductivity [8]. Heteroepitaxial or textured growth of diamond has been investigated to prepare large-area, high-quality, possibly single-crystalline diamond films [9].

Growth of textured or highly oriented diamond surfaces has been conducted via a variety of methods, for instance, by bias-enhanced nucleation processes [10,11,12], evolutionary growth processes [13,14], and processes exploiting epitaxy [15]. In particular, multi-step processes have been shown to yield highly {100}-textured diamond and larger crystals with the possibility to attain crystal coalescence [9]. Yet, the growth rate of the {100}-textured diamond is typically low, with values around 1 μm/h or lower [16,17,18,19], and the texture of the coating changes when growing a large area [17]. In regard to growth rate, Weng et al. obtained a growth rate of around 5 µm/h for {100}-textured diamond made by MPCVD [20], albeit the crystal size was between 1 and 10 µm, while the authors did not mention the area of homogeneous growth achieved [20]. Regarding diamond wafer area, Flöter et al. reported that the homogenous textured areas were around 20 cm^2^ [21], while most samples of the homogenous textures were much smaller [19]. Further, in the study of Flöter et al., the authors obtained diamond crystals with changing size from the center (20 µm) to the rim (1–3 µm) of the diamond plate, associated with lower plasma density away from the center of the sample [21]. These low crystallite sizes and the gradual change in crystallite size are expected to impact the thermal conductivity of the diamond plate.

In this study, a 3-inch homogeneous {100}-textured diamond was grown with growth rates exceeding 5 μm/h on a silicon wafer. The 3-inch homogeneous {100}-textured diamond growth was achieved by a combination of self-assembly seeding, yielding high nucleation densities, MPCVD growth at high vol% methane, and initially, in the presence of nitrogen to obtain the surface texture, followed by growth without nitrogen. After the detachment of the diamond film from the silicon substrate, a ≥0.8 mm thick free-standing diamond wafer with a 3-inch homogeneous {100} diamond surface texture and homogeneous crystallite size was obtained. The development of the texture was discussed in terms of the evolutionary growth mechanism. Finally, the thermal conductivity of the diamond wafer was determined and compared to that of a diamond wafer with similar thickness but without a particular texture.

## 2. Experimental

### 2.1. Surface Pre-Treatment

The substrate used in this experiment was a commercially available single-sided polished p-type (100) silicon wafer (with a diameter of 76.2 mm and a thickness of 3 mm, Hefei Kejing Materials Technology Co., Ltd., Hefei, China). The silicon wafer was pre-treated by a two-step procedure, comprising cleaning and seeding. (1) Consecutive ultrasonic cleaning in acetone (≥95.5%, Guangdong Guangshi Reagent Technology Co., Ltd., Zhaoqing, China) for 15 min, in deionized water (18.2 MΩ·cm, KLDS-100L, Chengdu Tangshi Kangning Technology Development Co., Ltd., Chengdu, China) for 5 min, and in ethanol (≥99.7%, ZKH Industrial Products Supermarket Co., Ltd., Shanghai, China) for 15 min, followed by drying with a nitrogen stream. (2) Ultrasonic seeding of the silicon wafer in an aqueous solution containing nanodiamond particles (5 nm particles, 10 mg/mL, ADAMAS, positive zetapotential, Adámas Nanotechnologies, Raleigh, NC, USA) for 15 min, followed by careful blow-drying with nitrogen. This seeding leads to a seeding density of ≥10^11^ particles/cm^2^, as assessed in a previous publication [22].

### 2.2. Diamond Growth

The diamond growth was conducted using a self-made microwave plasma chemical vapor deposition (MPCVD) machine with a power rating of 10 kW. The frequency of the MPCVD was 2.45 GHz. The gas sources were CH_4_, H_2_, and N_2_ with purities of 99.9999%. The volume percent (vol%) of N_2_ was 0.5 vol% (N_2_/H_2_). For diamond growth, the pressure was maintained at (12 ± 0.2) kPa and the power at (9000 ± 200) W. The flow rates were 400 sccm of hydrogen, 20 sccm of methane, and 1 sccm of nitrogen. For the initial 12 h, nitrogen was introduced into the growth gas composition. Afterwards, growth was continued for 186 h in a pure hydrogen and methane gas mixture. The temperature on the substrate sample was controlled with an infrared radiation thermometer. The detailed deposition parameters are listed in Table 1. Note that the power and pressure were slightly changed in order to keep the sample temperature constant.

For comparison, a diamond wafer was grown with continuous addition of 1 sccm nitrogen for 180 h. All other deposition parameters were broadly maintained.

The uniformity of the as-grown non-textured and highly textured samples was calculated to be ca. 11% and 13%. After lapping, the uniformity could be controlled to be even lower.

uniformity = (max value − min value)/(2 × average value) × 100%

### 2.3. Analysis

**Figure 1:**
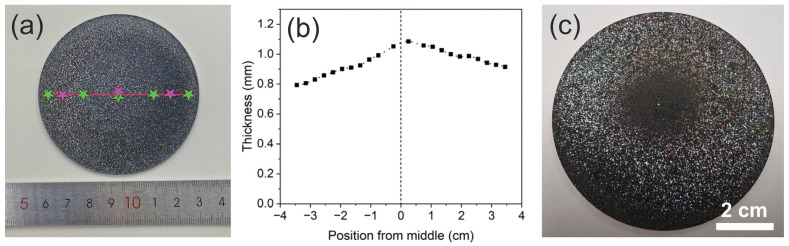
(**a**) Photograph of the 3-inch (7.5 cm diameter) polycrystalline diamond wafer made with the parameters stated in Table 1. The image shows the locations where Raman and optical microscopy (green stars) and XRD (purple stars) were measured. (**b**) The thickness of the diamond wafer shown in (**a**) is dependent on the position on the wafer. The number 0 denotes the center of the wafer. (**c**) Photograph of the 3-inch (7.5 cm) diamond wafer grown for 180 h without cessation of the nitrogen addition. Growth was achieved with the same parameters as described in the initial step in Table 1.

After growth of the diamond thick coating, the diamond was separated from the silicon substrate by a mixture of hydrofluoric acid (48 wt%, Sigma Aldrich, St. Louis, MO, USA) and nitric acid (65 wt%, Sigma Aldrich, St. Louis, MO, USA) in a ratio of 3:1, resulting in the 3-inch free-standing diamond wafer shown in Figure 1a. The thickness of these discs was measured with a caliper. The growth rate of the diamond wafer during CVD was determined by the thickness of the wafer divided by the growth time. Confocal Raman spectroscopy was conducted with the Renishaw (Horiba, Hamamatsu, Japan) plc/invia using a laser (RL532-08/100 mW, Renishaw (Horiba, Hamamatsu, Japan)) with an excitation wavelength of 532 nm. The spectra were taken with an 1800 diffraction grating, and measurements were averaged three times. Measurements were taken from five different locations on the 3-inch (7.5 cm) substrate along a line, namely, close to the edge (distance to edge less than 0.5 cm), at a distance of ca. 1.9 cm from the edge, close to the center of the sample, at a distance of ca. 1.9 cm away from the opposite edge, and close to the opposite edge, as indicated with the green stars in Figure 1b. At similar locations, the morphology of the substrate was assessed by optical microscopy (Soptop RX50M, SunGrant Suzhou Jingtong Instrument Co., Ltd., Suzhou, China) with a 10× air objective. The crystal structure of the samples was analyzed by X-ray diffraction (XRD, Empyrean, Panalytical, Worcestershire, UK) using Cu K_α_ radiation. The measurement was conducted for 20° ≤ 2Θ ≤ 120° at the three locations along a line, indicated in Figure 1b with the purple stars. The pole density was measured on a PANalytical Empyrean (Worcestershire, UK) X-ray diffractometer using Cu K_α_ as the X-ray source, with the χ-φ-Z (chi-phi-Z) sample stage and the PIXcel 3D detector. The in-plane (parallel to the surface) thermal diffusivity was measured via the laser flash method with the LFA 467 HyperFlash (Netzsch, Selb, Germany), equipped with a Xenon flash lamp and a HgCdTe (MCT) detector (Netzsch, Selb, Germany). Detection parameters were a (laser) pulse width of 40 to 50 ms, a laser voltage of 250 V, and a detection area of 17.5 mm. Prior to measurement, the nucleation side was lapped and polished. The in-plane thermal diffusivity, *α*(*T*)_‖_, was converted into the in-plane thermal conductivity, *λ*(*T*)_‖_, by Equation (1), using the heat capacity at constant pressure (*c_p_*) of diamond of 0.54 J/(g·K) (*c_p_* and the heat capacity at constant volume, *c_V_*, are for solids approximately the same) and the density of diamond, *ρ*. The latter is known to be 3500 kg·m^−3^ for single crystal diamond and the same for diamond grown via CVD methods.

(1) $${\lambda \left(T\right)}_{\|}={\alpha \left(T\right)}_{\|}*c_{p}\left(T\right)*\rho \left(T\right)$$

## 3. Results and Discussion

### 3.1. Diamond Wafer Appearance and Growth Rate

Initially, the cleaned silicon substrate was homogeneously seeded by nanodiamonds with seeding densities exceeding 10^11^ cm^−2^, according to a procedure outlined in reference [22]. The high seeding density arises from the electrostatic self-assembly seeding procedure and is conducive to homogeneous growth of the diamond wafer. The orientations of the crystal planes of the nanodiamond seeds (i.e., azimuthal and pole angles of crystal planes) are in contrast to the random orientations of the BEN and template methods. Initially, the film was grown for 12 h in a methane, hydrogen, and nitrogen (0.5% in hydrogen) gas mixture. These growth parameters and the addition of nitrogen are known to favor the growth of the (100) crystal facets and to be conducive to the growth rate of diamond coatings [23]. After this initial growth, the addition of nitrogen in the CVD chamber was stopped while the other deposition parameters were maintained, and the growth continued for 186 h. The change in gas chemistry resulted in a reduction in the quotient of growth rates of (100) and (111) crystal facets compared to the case with nitrogen addition. This is important for the crystal texture, as discussed in Section 3.3. Furthermore, limiting the incorporation of nitrogen is known to be beneficial to maintaining good optical and thermal properties of diamond, as doping with nitrogen is detrimental in regard to these properties [24].

The optical photograph, shown in Figure 1a, signifies the homogeneity of the diamond coating. The thickness of the diamond wafer across the line shown in Figure 1a is depicted in Figure 1b. The lowest thickness was measured close to the rim with ca. 0.80 mm, while the highest thickness was measured close to the middle (position 0 cm) with ca. 1.09 mm (ca. 25% thicker). The thickness dwindles slightly and is nearly linear from the middle position, in agreement with the higher temperature and higher plasma density in the middle of the plasma ball. The thickness of the diamond wafer translates to growth rates of 4.0–5.5 µm/h. A diamond wafer grown at similar growth conditions, but at continuous addition of 1 sccm nitrogen yielded a diamond wafer with a thickness of 0.77–1.06 mm (grown for 180 h), translating to a growth rate of 4.28–5.88 µm/h, slightly higher than the here shown two step growth process, but the texture is not homogeneous across the whole surface area, as can be seen in Figure 1c. Post-processing by lapping can be used to reduce the thickness variation. Further, in situ, one can optimize the reactor design and microwave power distribution to homogenize plasma density across the wafer surface, as well as optimize the structure of the molybdenum holder to achieve a more uniform growth rate [25].

Nitrogen is well-known to increase the growth rate of diamond [26,27,28], albeit it reduces the crystal quality, specifically for optical and thermal properties [24]. In this study, the increase in growth rate was negligible and of little importance; rather, the importance of the nitrogen addition during initial growth was related to the development of the diamond texture. Note that the growth rates stated here are at least 4 times that of most MPCVD- and HFCVD-made {100}-textured diamond coatings and wafers, often prepared at growth rates of close to or less than 1 µm/h [16,17,18,19]. Weng et al. obtained a growth rate of around 5 µm/h for {100}-textured diamond made by MPCVD on a 3-inch substrate [20], yet no comment on the homogeneity of the texture was made by the authors. High growth rates have been showcased by Yang et al. for ˂110˃ diamond on a 1.5-inch substrate, but the homogeneity of the texture was not discussed [29]. Note that higher growth rates for polycrystalline and single crystal diamond have been reported [30,31,32,33,34].

Previous studies have shown different surface morphologies and texturing in the center, annulus, and rim [17]. The varied morphology and texture have been proposed to stem from a very narrow deposition parameter window for the textured growth of diamond [35,36]. Difficulty in growing homogeneous {100}-textured diamond was also observed in this study if a two-step process was not followed. Continuously supplying 1 sccm nitrogen to the reactive gas mixture during growth for 180 h leads to the formation of different textures along the substrate surface, as indicated in Figure 1c. In the center of the sample, a dark rotational symmetric circular (diameter ca. 2 cm) region can be gleaned, which is dominated by (111) pyramidal facets. In contrast, at the rim of the sample (outer 2 cm), the diamond wafer appears brighter, which is assigned to the {100} texture according to microscopy and XRD. Change in texture is likely related to the variations in the α value arising from changes in growth gas composition and temperature at the substrate, as both parameters are known to affect the texture; the former has been suggested to have the stronger effect on the texture [14].

### 3.2. Homogeneity of the {100} Texture

**Figure 2:**
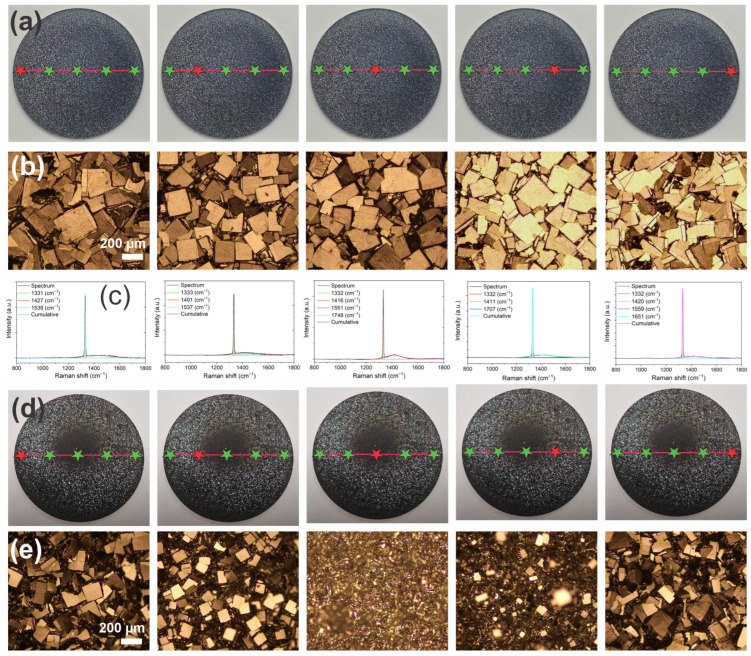
(**a**) Photograph of the freestanding homogeneous {100}-textured diamond wafer made with the two-step procedure indicated in Table 1, indicating with a red star the location of measurement of (**b**) optical microscopy images and (**c**) Raman spectra. While the red stars indicate the current measurement location, the green stars indicate other measurement positions of the series of measurements. (**d**) Photograph of the 3-inch diamond wafer grown for 180 h with continuous nitrogen addition, indicating the measurement locations of the (**e**) optical microscopy images.

In order to ascertain the homogeneity of the {100}-textured diamond wafer, spatially resolved optical microscopy images and Raman spectra were taken. Figure 2a shows the measurement locations, and the corresponding microscopy images and Raman spectra are shown in Figure 2b,c. The microscopy images highlight the presence of a {100}-textured surface. The crystals have a grain size of ca. 210 ± 60 µm. This crystal size is larger than most textured diamond films, which are on the order of a few µm [13,19,23,37,38], unless highly oriented diamond growth leading to coalescence was attained [9,37]. A reduction in grain boundaries and an increase in crystal size are envisaged to improve thermal, electrical [37], and optical properties of the diamond wafer. Although the diamond (100) crystal face is oriented parallel to the sample surface, the azimuthal orientation is random, which stems from the random orientation of the nanodiamond seeds prior to growth and the evolutionary growth mechanism. The Raman spectra in Figure 2c signify the growth of diamond with high purity, with sharp peaks at ca. 1332.0 cm^−1^, which refers to the zone-center phonon (ZCP) peak of diamond. The ZCP peak position does not vary much with measurement location. However, the intensity of non-diamond phases around 1450 cm^−1^, i.e., suggested to stem from the ν_3_ mode of trans polyacetylene (controversial) [39,40], increases slightly in the center of the wafer.

For comparison of the texture, a diamond wafer was grown with continuous addition of 1 sccm nitrogen for 180 h, shown in Figure 1c and Figure 2d. The texture of this sample is illustrated in the optical microscopy images shown in Figure 2e. In the circular center region, the {100} texture is virtually absent, as evidenced by the microscopy image dominated by small grains and likely (111) pyramids. At the rim, {100} texture is observed, and the crystal size is 150 ± 50 µm, smaller than the sample in Figure 2a. Some crystals appear to have a large tilt angle, although this is difficult to elicit from optical microscopy images. The second and fourth optical microscopy images in Figure 2e signify that the texture of the diamond wafer is gradually changing radially from the center of the wafer, from a {111}-dominated texture to a {100}-dominated texture at the rim. During this gradual change, the size of the {100} diamond grains gradually increased.

To investigate the homogeneity of the “homogeneously” {100}-textured diamond wafer further, the diamond quality and the full width half maximum (FWHM) of the diamond peak were determined. The diamond quality *f_q_* in percent was determined by the ratio of the Raman area of the diamond phase (*I_dia_*) to the sum of Raman areas of the diamond phase and all non-diamond phases (*I_non-dia_*), as shown in Equation (2) [41,42]. The factor 75 originates from the greater Raman efficiency of carbon sp^2^ phases compared to carbon sp^3^ (5145 Å laser excitation) [43]. As the factor depends on the excitation wavelength, the calculation here is an approximation.

(2) $$f_{q}=100\left(\frac{75*I_{dia}}{75*I_{dia}+\sum I_{non-dia}}\right)$$

**Figure 3:**
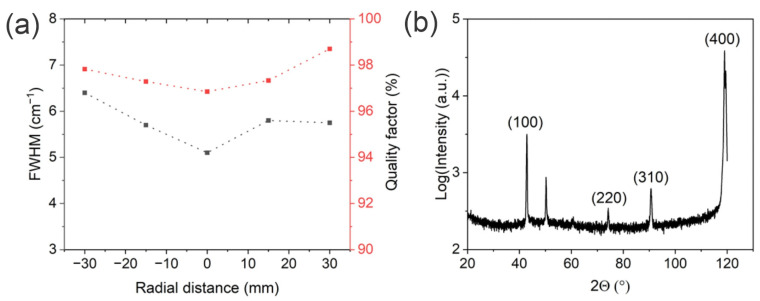
(**a**) Full-width half maximum (FWHM) and quality factor of the {100}-textured diamond wafer determined from the spectra given in Figure 2c. (**b**) Representative XRD pattern of the diamond wafer (y-axis in log-scale).

Figure 3a shows the FWHM and the quality factor for the five different positions on the diamond wafer. The FWHM increases only slightly from ca. 5 cm^−1^ at the center to ca. 6.5 cm^−1^ at 3 cm away from the center. Simultaneously, the quality factor is between 97% and 99% for the whole diamond wafer. In contrast, diamond quality for a wafer grown for the whole growth process with nitrogen varies between 88 (center) and 96% (rim). This reduction in diamond quality is a result of increased non-diamond carbon content in the deposit [24]. Figure 3b shows a representative XRD diffraction pattern obtained for the diamond wafer. Two prominent crystal facets can be discerned, namely, (111) at 2Θ of 42.8° and (400) at 2Θ of 118.9°. The second peak at 119.4° arises from the *K*α_2_ radiation of the copper diffracted at the (400) facet. Further, minute reflections of facets at 2Θ of 74.2° and 90.5° assigned to (220) and (311) crystal facets of diamond, respectively, can be discerned. The XRD patterns at all three measurement locations are dominated by the (400) diffraction peak, indicating highly homogeneous {100}-textured diamond. The crystal facets at the surface are (100) while the grain boundaries and grooves are dominated by the (111) crystal face [37]. The preferred (100) texture of the diamond wafer can be substantiated by the intensity ratio of I_(400)_/I_(111)_ obtained from the XRD spectra. Values between 5 and 18 were obtained (average 11.8 ± 6.5).

### 3.3. Growth Mechanism of the {100} Texture

Initially, the nanodiamond seeds are oriented randomly. The origin of the preferred growth of {100}-textured diamond is related to the evolutionary selection during CVD growth, first introduced by van der Drift in 1966 [44] (and 1967 [45]). In this evolutionary selection theory, the fast-growing diamond facets overgrow the slower ones. In this study, the addition of nitrogen has a grave effect on the growth factor α [13,27], defined by the growth rates on the {100} (V_100_) and {111} (V_111_) faces, according to Equation (3) [14].

(3) $$\alpha =\sqrt{3}\cdot \frac{\left(V_{100}\right)}{\left(V_{111}\right)}$$

**Figure 4:**
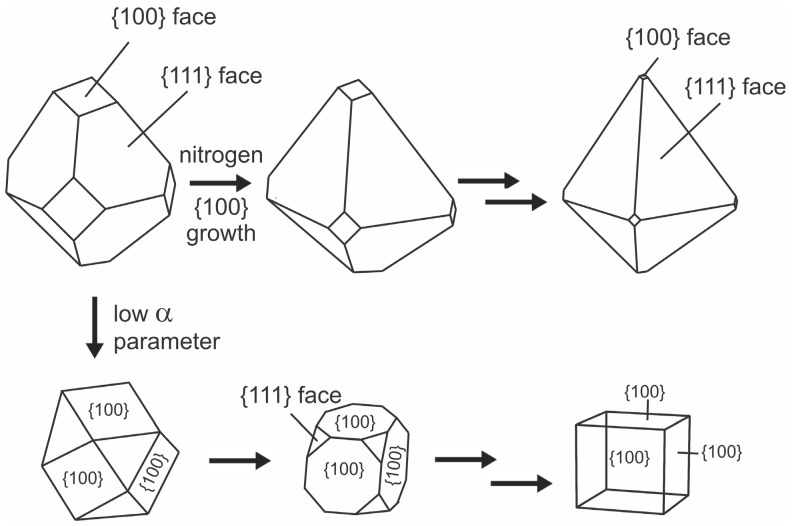
Schematic illustration of the growth of the {100} and {111} faces. For a high growth parameter alpha (nitrogen addition, top row), the {100} faces grow the fastest, resulting in dying out. For a low growth parameter alpha (bottom row), the {100} crystal facets dominate. Drawn after ref. [49].

Indeed, nitrogen encourages the formation of {100} facets compared to {111} and {110} [27,46]. A growth factor of close to 3 or above results in the fast growth of (100) compared to (111) crystal facets [14]. Simultaneously, the crystal facets perpendicular to the substrate surface grow higher than crystal facets with a considerable angle, and these misoriented crystal facets are buried (overgrown) in the second deposition process (without nitrogen addition). The reason for choosing this process is as follows. During growth at high α values, growth is fastest in the ˂100˃ facet direction and the (100) surfaces diminish (die out) while (111) pyramids are often obtained [14,18], as shown in Figure 4. To circumvent this, either deposition at values slightly lower than an alpha growth factor of 3 can be done (this process is difficult to control, especially for large surface areas) or multi-step deposition processes can be conducted, where at first the α value is close to 3, and later, the growth factor is reduced [9,13,14,18,37,47,48]. In this study, the growth factor was initially high due to the introduction of nitrogen, and was reduced due to the cessation of the introduction of nitrogen gas into the CVD chamber. Due to the shape of the crystal, the α value can be estimated to be close to 1 [49], and the result is the cubic form of the grown crystals, as shown in Figure 4 [14]. The combination of the higher initial growth rate of the (100) facets and the higher growth rate of (111) facets after 12 h results in the overgrowth of the initially slower-growing crystal (111) facets and misaligned (100) faces. Finally, the (111) crystal facets are only located at the grain boundaries, while the cuboid diamond crystals prominently feature the (100) crystal plane close to parallel to the substrate surface. Due to the long growth time without the addition of nitrogen, the diamond crystals grow larger (in-plane, due to the high growth rate of the (111) facets), but the coalescence of the diamond crystals is hindered by the in-plane misorientation of the crystals. The homogeneity of the texture stems likely from the large deposition parameter window at α ≤ 1 and β ≤ 1 [49], where β is defined by relative displacement speeds of the (100) and (110) crystal facets (Equation (4)) while the pre-factor stems from the square root of the sum of the squares of the Miller indices of their respective planes [49]. If the beta parameter is larger than this value, diamond crystals with mixed {100} and {110} facets would grow, as discussed by Silva et al. [49].

(4) $$\beta =\sqrt{2}\cdot \frac{\left(V_{100}\right)}{\left(V_{110}\right)}$$

**Figure 5:**
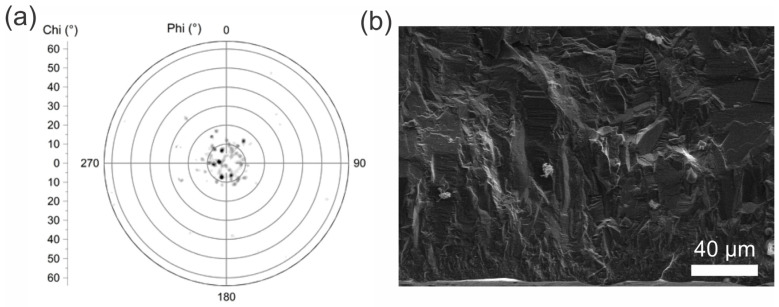
(**a**) XRD pole figure of {400}-textured diamond. (**b**) SEM cross-section of sample SN. Bottom shows the nucleation site with a smaller crystallite size.

The {400} pole density is stereographically illustrated in Figure 5a to estimate the deviation of the {100}-texture from being parallel to the growth surface. It is evident from the graph that the textured diamond film has a misaligned tilt angle *χ* of ≤15° in relation to the substrate surface, with most of the crystal facets having a misaligned tilt angle of less than 10°, yet it is not as low as epitaxially grown diamond or diamond nucleated with bias assistance. Further, there is no azimuthal orientation of the crystal as observed in the microscopy images. This is also confirmed by the random distribution observed in the {400} pole density. The misalignment angle *χ* is across the whole sample, signifying the homogeneity of the {100} texture.

### 3.4. Thermal Conductivity

Finally, the thermal conductivity of the {100}-textured diamond wafer was measured and compared to a wafer made without a texture (without any addition of nitrogen). The latter wafer had an in-plane thermal diffusivity of 452 mm^2^/s, translating to an in-plane (‖) thermal conductivity of 854 W/(mK). In contrast, the in-plane thermal diffusivity and thermal conductivity for the textured diamond wafer were 595 mm^2^/s and 1125 W/(mK), respectively. The higher thermal conductivity for the textured diamond wafer arises from the larger crystal size and higher crystal quality (less non-diamond deposits) [24,50]. Both parameters are well-known to affect the thermal conductivity considerably [24,51,52]. For instance, the crystal size affects the thermal conductivity due to increased phonon scattering at the grain boundaries [51,52], and this scattering becomes more pronounced when approaching the phonon mean free pathlength [53]. The obtained values are less than single crystal diamond [54], but in agreement with polycrystalline diamond [54,55]. The addition of nitrogen during the initial growth leads to deterioration of thermal conductivity, i.e., due to phonon scattering at impurity atoms and lower diamond quality. Furthermore, polycrystalline CVD diamond features large anisotropy in thermal conductivity arising from diamond quality and crystal size gradients across the diamond film thickness, namely, small crystallites with low diamond quality (low thermal conductivity—due to boundary scattering) are formed at the nucleation site, while these crystals often grow with increasing film thickness if renucleation is suppressed [56]. In this paper, the nucleation side was lapped and polished, which removes a few micrometers of the plate. This is mainly done to enhance the uniformity and simultaneously increase overall thermal conductivity as the small crystallites (Figure 5b) and low diamond quality close to the nucleation site decrease phonon mean free path and increase phonon scattering [56]. For these samples, these processes also removed the nitrogen-doped region of the diamond plate, thereby improving thermal conductivity considerably.

Notably, the thermal conductivity is anisotropic, and the cross-plane (vertical, ⊥) thermal conductivity is known to be higher due to the columnar structure of CVD-grown diamond (observable in Figure 5b), resulting in less phonon scattering at grain boundaries [50,55]. The value for the ⊥ thermal conductivity is often 10 to 50% higher than that for the ‖ thermal conductivity [57], depending on the grain size and growth mode, i.e., columnar growth structure. Here, a conservative estimate of 10 to 30% was used to estimate the ⊥ thermal conductivity, resulting in ⊥ thermal conductivities of 939–1110 and 1237–1462 W/(mK) for the untextured and textured diamond wafer, respectively, approaching the thermal conductivity of single crystal diamond (ca. 2400 W/(mK)) [4].

## 4. Conclusions

In summary, we have demonstrated that {100}-textured diamond thick films on silicon (100) can be grown by a two-step MPCVD process, achieving surface areas in excess of 175 cm^2^. The initial growth of diamond in the presence of nitrogen favors the growth of (100) crystal facets over (111) and (110), and a growth parameter α of ca. 3 is obtained [37]. In the second growth step, addition of nitrogen was ceased, and the growth parameter is starkly reduced, resulting in overgrowth of misoriented (100)-diamond facets and (111) and (110) facets, according to the evolutionary selection theory by van der Drift [45]. This process results in the preferential formation of the {100}-textured diamond film and allows the large area deposition. Less affected by phonon scattering at the grain boundaries, the horizontal thermal conductivity is 1125 W/(mK) larger than the untextured microcrystalline diamond (854 W/(mK)). The established process for large area {100}-textured diamond free-standing wafers is interesting for the fabrication of heat spreaders for CPU, GPU, HEMTs, and high-power light-emitting diodes.

## Figures and Tables

**Table 1:** Deposition parameters used for the growth of the {100}-textured diamond wafer on <100> Si.

H_2_/CH_4_ Gas Flow (sccm)	400/20
Additional gas	first 12 h: 1 sccm N_2_
Sample temperature (°C)	900–920
Applied microwave power (kW)	8.5–9.2
Deposition pressure (kPa)	11.8–12.0
Deposition time (h)	198
Thickness (mm)/growth rate (µm/h)	0.80–1.09/4.0–5.5
I_400_/I_111_ *	11.8 ± 6.5

* This is the intensity ratio of the XRD diffraction peaks for the (400) and the (111) crystal facets of diamond, determined from their respective peak areas (XRD diffraction peaks).

## Data Availability

The original contributions presented in this study are included in the article. Further inquiries can be directed to the corresponding authors.

## References

[1] Zulkharnay R., May P.W. (2024). Applications of diamond films: A review. Funct. Diam..

[2] Ding Y., Li J., Hao Z., Wang Q., Zhang H., Peng Y., Chen M. (2024). Enhanced Heat Dissipation of High-Power InGaN Blue Laser Diode Through Diamond Substrates. IEEE Photonics Technol. Lett..

[3] Handschuh-Wang S., Xing Z., Wang T. (2026). Structuring and patterning approaches for diamond—Toward the third dimension. Mater. Today Phys..

[4] Inyushkin A.V., Taldenkov A.N., Ralchenko V.G., Bolshakov A.P., Koliadin A.V., Katrusha A.N. (2018). Thermal conductivity of high purity synthetic single crystal diamonds. Phys. Rev. B.

[5] Dong H., Wen B., Melnik R. (2014). Relative importance of grain boundaries and size effects in thermal conductivity of nanocrystalline materials. Sci. Rep..

[6] Efimov V.B., Mezhov-Deglin L.P. (1999). Phonon scattering in diamond films. Phys. B.

[7] Schreck M., Asmussen J., Shikata S., Arnault J.-C., Fujimori N. (2014). Large-area high-quality single crystal diamond. MRS Bull..

[8] Wolter S.D., Borca-Tasciuc D.A., Chen G., Govindaraju N., Collazo R., Okuzumi F., Prater J.T., Sitar Z. (2003). Thermal conductivity of epitaxially textured diamond films. Diam. Relat. Mater..

[9] Jiang X., Fryda M., Jia C.L. (2000). High quality heteroepitaxial diamond films on silicon: Recent progresses. Diam. Relat. Mater..

[10] Wolter S.D., Stoner B.R., Glass J.T., Ellis P.J., Buhaenko D.S., Jenkins C.E., Southworth P. (1993). Textured growth of diamond on silicon via in situ carburization and bias-enhanced nucleation. Appl. Phys. Lett..

[11] Maeda H., Ohtsubo K., Irie M., Ohya N., Kusakabe K., Morooka S. (1995). Determination of diamond [100] and [111] growth rate and formation of highly oriented diamond film by microwave plasma-assisted chemical vapor deposition. J. Mater. Res..

[12] Tachibana T., Hayashi K., Kobashi K. (1996). Azimuthal rotation of diamond crystals epitaxially nucleated on silicon {001}. Appl. Phys. Lett..

[13] Locher R., Wild C., Herres N., Behr D., Koidl P. (1994). Nitrogen stabilized 〈100〉 texture in chemical vapor deposited diamond films. Appl. Phys. Lett..

[14] Wild C., Koidl P., Müller-Sebert W., Walcher H., Kohl R., Herres N., Locher R., Samlenski R., Brenn R. (1993). Chemical vapour deposition and characterization of smooth {100}-faceted diamond films. Diam. Relat. Mater..

[15] Yang G., Lu Y., Wang B., Xia Y., Chen H., Song H., Yi J., Deng L., Wang Y., Li H. (2022). Chemical Vapor Deposition of <110> Textured Diamond Film through Pre-Seeding by Diamond Nano-Sheets. Materials.

[16] Chang T.-F., Chang L. (2001). Highly oriented diamond growth on positively biased Si substrates. J. Mater. Res..

[17] Cao G.Z., Schermer J.J., van Enckevort W.J.P., Elst W.A.L.M., Giling L.J. (1996). Growth of {100} textured diamond films by the addition of nitrogen. J. Appl. Phys..

[18] Kawarada H., Suesada T., Nagasawa H. (1995). Heteroepitaxial growth of smooth and continuous diamond thin films on silicon substrates via high quality silicon carbide buffer layers. Appl. Phys. Lett..

[19] Delfaure C., Tranchant N., Mazellier J.-P., Ponard P., Saada S. (2016). Monitoring texture formation during diamond growth by specular and diffuse reflectance interferometry. Diam. Relat. Mater..

[20] Weng J., Wang J.H., Dai S.Y., Xiong L.W., Man W.D., Liu F. (2013). Preparation of diamond films with controllable surface morphology, orientation and quality in an overmoded microwave plasma CVD chamber. Appl. Surf. Sci..

[21] Flöter A., Güttler H., Schulz G., Steinbach D., Lutz-Elsner C., Zachai R., Bergmaier A., Dollinger G. (1998). The nucleation and growth of large area, highly oriented diamond films on silicon substrates. Diam. Relat. Mater..

[22] Xing Z., Handschuh-Wang S., Wang T., Han P., He B. (2025). Controlled seeding density of nanodiamonds on silicon and its influence on diamond film adhesion. Funct. Diam..

[23] Ayres V.M., Bieler T.R., Kanatzidis M.G., Spano J., Hagopian S., Balhareth H., Wright B.F., Farhan M., Abdul Majeed J., Spach D. (2000). The effect of nitrogen on competitive growth mechanisms of diamond thin films. Diam. Relat. Mater..

[24] Bachmann P.K., Hagemann H.J., Lade H., Leers D., Wiechert D.U., Wilson H., Fournier D., Plamann K. (1995). Thermal properties of C/H-, C/H/O-, C/H/N- and C/H/X-grown polycrystalline CVD diamond. Diam. Relat. Mater..

[25] Handschuh-Wang S., Liu X., He B., Han P., Jiang X., Wang T. (2026). Interfacial Engineering for Enhanced Adhesion of Diamond Coatings. Small.

[26] Oberg L.M., Batzer M., Stacey A., Doherty M.W. (2021). Nitrogen overgrowth as a catalytic mechanism during diamond chemical vapour deposition. Carbon.

[27] May P.W., Zulkharnay R. (2025). Diamond thin films: A twenty-first century material. Part 2: A new hope. Philos. Trans. R. Soc. A Math. Phys. Eng. Sci..

[28] Jin S., Moustakas T.D. (1994). Effect of nitrogen on the growth of diamond films. Appl. Phys. Lett..

[29] Yang G., Sun P., Zhu T., Wang Y., Li S., Liu C., Yang G., Yang K., Yang X., Lian W. (2024). Fabrication, microstructure and optical properties of 〈110〉 textured CVD polycrystalline diamond infrared materials. Diam. Relat. Mater..

[30] Bolshakov A.P., Ralchenko V.G., Yurov V.Y., Shu G., Bushuev E.V., Khomich A.A., Ashkinazi E.E., Sovyk D.N., Antonova I.A., Savin S.S. (2019). Enhanced deposition rate of polycrystalline CVD diamond at high microwave power densities. Diam. Relat. Mater..

[31] Ren Z., Wang C., Zhang J., Zhu Z., Su K., Man W., Fu Y., Chen J., Li J., Zhu W. (2025). Expansion growth of <110>-oriented single crystal diamond. Appl. Surf. Sci..

[32] Ohmagari S. (2023). Single-crystal diamond growth by hot-filament CVD: A recent advances for doping, growth rate and defect controls. Funct. Diam..

[33] Ren Y., Li X., Lv W., Dong H., Cheng Q., Yue F., Wöhrl N., Mendes J.C., Yang X., Li Z. (2024). Recent progress in homoepitaxial single-crystal diamond growth via MPCVD. J. Mater. Sci. Mater. Electron..

[34] Bolshakov A.P., Ralchenko V.G., Shu G., Dai B., Yurov V.Y., Bushuev E.V., Khomich A.A., Altakhov A.S., Ashkinazi E.E., Antonova I.A. (2020). Single crystal diamond growth by MPCVD at subatmospheric pressures. Mater. Today Commun..

[35] Schermer J.J., de Theije F.K. (1999). Nitrogen addition during flame deposition of diamond: A study of nitrogen-enhanced growth, texturing and luminescence. Diam. Relat. Mater..

[36] Gu C., Jin Z., Wang C., Zou G., Sakamoto Y., Takaya M. (1998). Growth of (100) orientation diamond film deposited by MWPCVD methods using the gaseous mixtures of CH_4_, CO and H_2_. Diam. Relat. Mater..

[37] Janischowsky K., Stammler M., Stöckel R., Ley L. (1999). Growth of high quality, large grain size, highly oriented diamond on Si (100). Appl. Phys. Lett..

[38] Chen K., Tao T., Hu W., Ye Y., Zheng K., Ye J., Zhi T., Wang X., Liu B., Zhang R. (2023). High-speed growth of high-quality polycrystalline diamond films by MPCVD. Carbon Lett..

[39] Ferrari A.C., Robertson J. (2001). Origin of the $1150\ensuremath{-}{\mathrm{cm}}^{\ensuremath{-}1}$ Raman mode in nanocrystalline diamond. Phys. Rev. B.

[40] Prawer S., Nemanich R.J. (2004). Raman spectroscopy of diamond and doped diamond. Philos. Trans. R. Soc. A Math. Phys. Eng. Sci..

[41] Izak T., Babchenko O., Varga M., Potocky S., Kromka A. (2012). Low temperature diamond growth by linear antenna plasma CVD over large area. Phys. Status Solidi B.

[42] Silva F., Gicquel A., Tardieu A., Cledat P., Chauveau T. (1996). Control of an MPACVD reactor for polycrystalline textured diamond films synthesis: Role of microwave power density. Diam. Relat. Mater..

[43] Wada N., Solin S.A. (1981). Raman efficiency measurements of graphite. Phys. B+C.

[44] van der Drift A. (1966). Texture of a vapour-deposited leadmonoxide layer. Philips Res. Rep..

[45] van der Drift A. (1967). Evolutionary selection, a principle governing growth orientation in vapor-deposited layers. Philips Res. Rep..

[46] Tung J.-C., Li T.-C., Teseng Y.-J., Liu P.-L. (2021). Effect of Nitrogen on the Growth of (100)-, (110)-, and (111)-Oriented Diamond Films. Appl. Sci..

[47] Janischowsky K., Stammler M., Ley L. (1999). High quality textured growth of oriented diamond thin films on Si (100) in a hot filament-CVD system. Diam. Relat. Mater..

[48] Suesada T., Nakamura N., Nagasawa H., Kawarada H. (1995). Initial Growth of Heteroepitaxial Diamond on Si(001) Substrates via β-SiC Buffer Layer. Jpn. J. Appl. Phys..

[49] Silva F., Bonnin X., Achard J., Brinza O., Michau A., Gicquel A. (2008). Geometric modeling of homoepitaxial CVD diamond growth: I. The {100}{111}{110}{113} system. J. Cryst. Growth.

[50] Sood A., Cho J., Hobart K.D., Feygelson T.I., Pate B.B., Asheghi M., Cahill D.G., Goodson K.E. (2016). Anisotropic and inhomogeneous thermal conduction in suspended thin-film polycrystalline diamond. J. Appl. Phys..

[51] Wang Y., Sun B. (2024). Anomalously strong size effect on thermal conductivity of diamond microparticles. Appl. Phys. Lett..

[52] Anaya J., Rossi S., Alomari M., Kohn E., Tóth L., Pécz B., Hobart K.D., Anderson T.J., Feygelson T.I., Pate B.B. (2016). Control of the in-plane thermal conductivity of ultra-thin nanocrystalline diamond films through the grain and grain boundary properties. Acta Mater..

[53] Tomabechi R., Taniguchi R., Kato H., Cho J., Hori T. (2025). Phonon mean free path analysis in polycrystalline nanostructured thin films. Int. J. Heat Mass Transf..

[54] Yamamoto Y., Imai T., Tanabe K., Tsuno T., Kumazawa Y., Fujimori N. (1997). The measurement of thermal properties of diamond. Diam. Relat. Mater..

[55] Zhang C., Vispute R.D., Fu K., Ni C. (2023). A review of thermal properties of CVD diamond films. J. Mater. Sci..

[56] Graebner J.E., Jin S., Kammlott G.W., Herb J.A., Gardinier C.F. (1992). Large anisotropic thermal conductivity in synthetic diamond films. Nature.

[57] Graebner J.E., Pan L.S., Kania D.R. (1995). Thermal Conductivity of Diamond. Diamond: Electronic Properties and Applications.

